# A Note on Co-Existent Infectious Diseases

**Published:** 1908-01-18

**Authors:** J. T. C. Nash


### A NOTE ON CO-EXISTENT INFECTIOUS DISEASES.

By J. T. C. NASH, M.D.

Concomitant infection, or the co-existence of two or more separate infectious diseases, occurs in about 2 per cent. of the cases admitted to hospital in large towns. In the Metropolitan Asylums Boards' hospitals during 1905, 430 cases of mixed infection occurred among 21,210 of scarlet fever, diphtheria, and enteric fever. Whooping-cough, measles, and chicken-pox are the usual co-existing diseases in reference to scarlet fever and diphtheria. In an appreciable percentage of cases scarlet fever and diphtheria co-exist, but in only a fractional percentage of cases do we witness the co-existence of enteric fever with scarlet fever or diphtheria.

In January 1906 I recorded * a case of co-existing infection with enteric fever and diphtheria, in which recovery in due time ensued. The following case is not without interest. A man aged 30 was admitted to hospital on a notification of enteric fever on November 8, 1906, with a definite history of oysters from a polluted source eaten within three weeks of the onset of illness, and exhibiting the classical symptoms and signs of typhoid fever, including deafness, pyrexia, enlarged spleen, characteristic rash, and typical stools. The blood next day gave a definite positive Widal reaction.

A note was made on admission of the very foul state of the patient's nose and mouth, the odour from which was penetrating. The face was dusky and the neck appeared swollen. The day after admission, in consequence of nursing attentions, he was cleaner about the nose and throat; but hoarseness and a laryngeal cough continued.

In the course of the next two days these symptoms became more pronounced, and on the following morning while I was at his bedside the patient coughed out a pellet which looked like a piece of diphtheritic membrane. On bacterioscopic examination of the expectorated pellet I found definite diphtheria bacilli. Diphtheria antitoxin was administered forthwith the same day. Any attempt to use a tongue depressor with a view to inspect the pharynx induced a violent expulsive cough.

Relief appeared to follow the use of antitoxin. The advisability of performing tracheotomy was considered but was deemed unnecessary in the absence of urgent obstructive respiratory symptoms. About 12 hours later, at 3 a.m., while in the act of coughing, the patient died before surgical aid could be summoned. The immediate cause of death was sudden spasm or obstruction of the glottis, the unfortunate patient rapidly turning black in the face according to the nurse in attendance. He was an alcoholic subject with a ten years' history of gross dissipation. He was suffering from an undoubted attack of typhoid fever, and died suddenly of glottic spasm or suffocation, the result of diphtheritic laryngitis.

The above facts indicate some difficulty as to the certification and classification of the death. The actual immediate cause of death was laryngeal obstruction presumably due to diphtheria, but in order of sequence the patient died suffering from:

(*a*) Chronic alcoholism (and a suggestion of syphilis),

(*b*) Undoubted enteric fever,

(*c*) Diphtheritic laryngitis.

It seems possible that some cases recorded as "laryngo-typhoid" might have really been of diphtheritic origin. In the absence of a bacteriological examination this would remain a moot point. Definite ulceration has been found in those parts of the larynx provided with adenoid tissue, and in some instances extensive perichondritis with necrosis and exfoliation of the cartilages has been observed. The typhoid bacillus has been looked for, but has not been found in these lesions. In the case here recorded bacteriological examination definitely established that the laryngeal affection was of true diphtheritic origin.

Dreschfeld, in his monograph on enteric fever in Allbutt's "System of Medicine" mentions only some 50 cases of this association of diphtheria with enteric fever, and comments on its extreme rarity.

Hitherto in fever hospital administration I have for years past had every case, whether admitted as one of scarlet fever or one of diphtheria, swabbed for examination for the bacteria present in the fauces on admission. The diphtheria bacillus is found in an appreciable percentage of scarlet fever admissions, and precautions can at once be taken to deal specially with the complication. Since I adopted this procedure post-scarlatinal diphtheria has been unknown in my hospital. The experience recorded in this note suggests the advisability of swabbing the throat of every patient on admission to a fever hospital, especially if any throat or nose trouble is apparent.

* The *Med. Press*, Jan. 31, 1906, p. 121.

